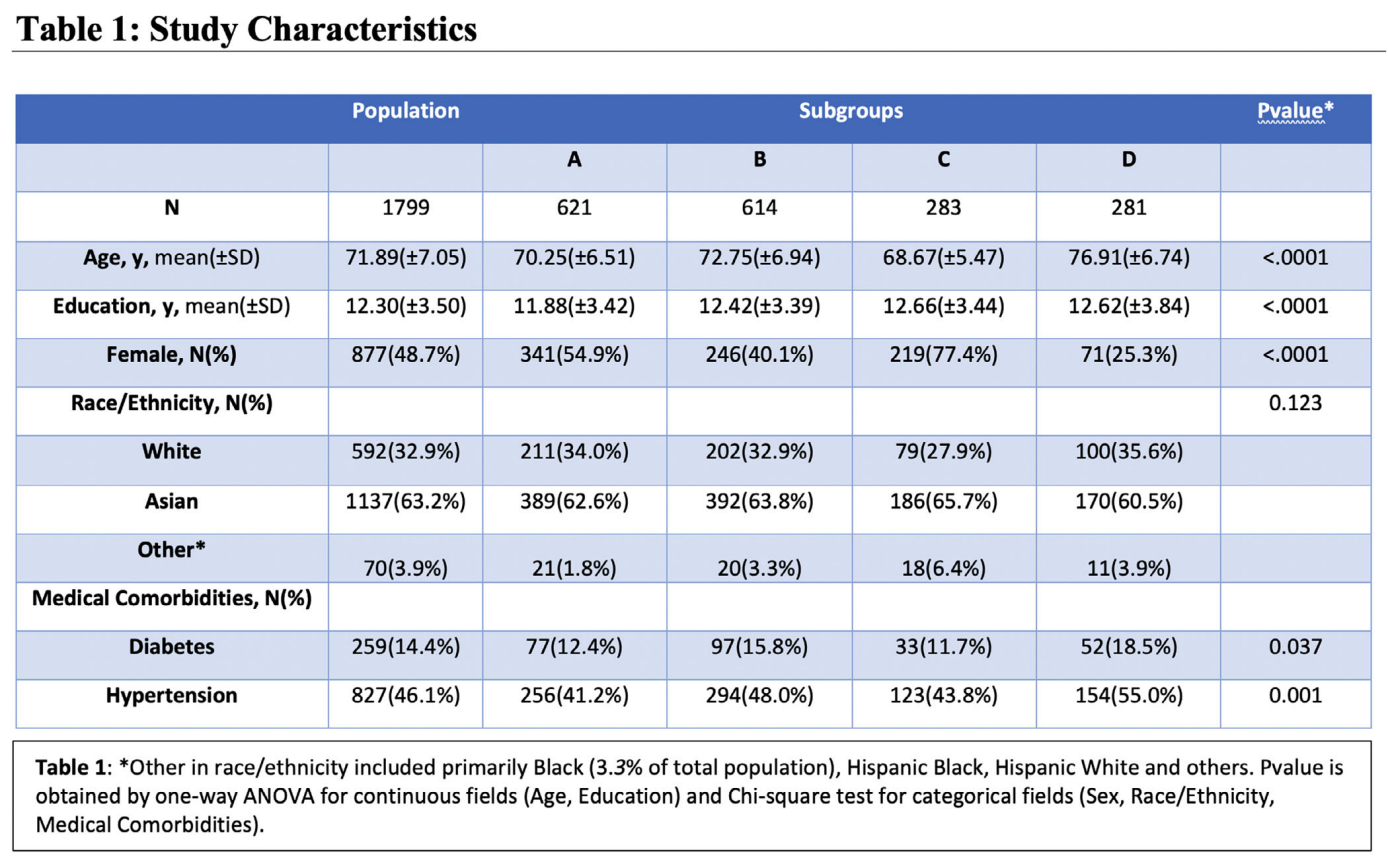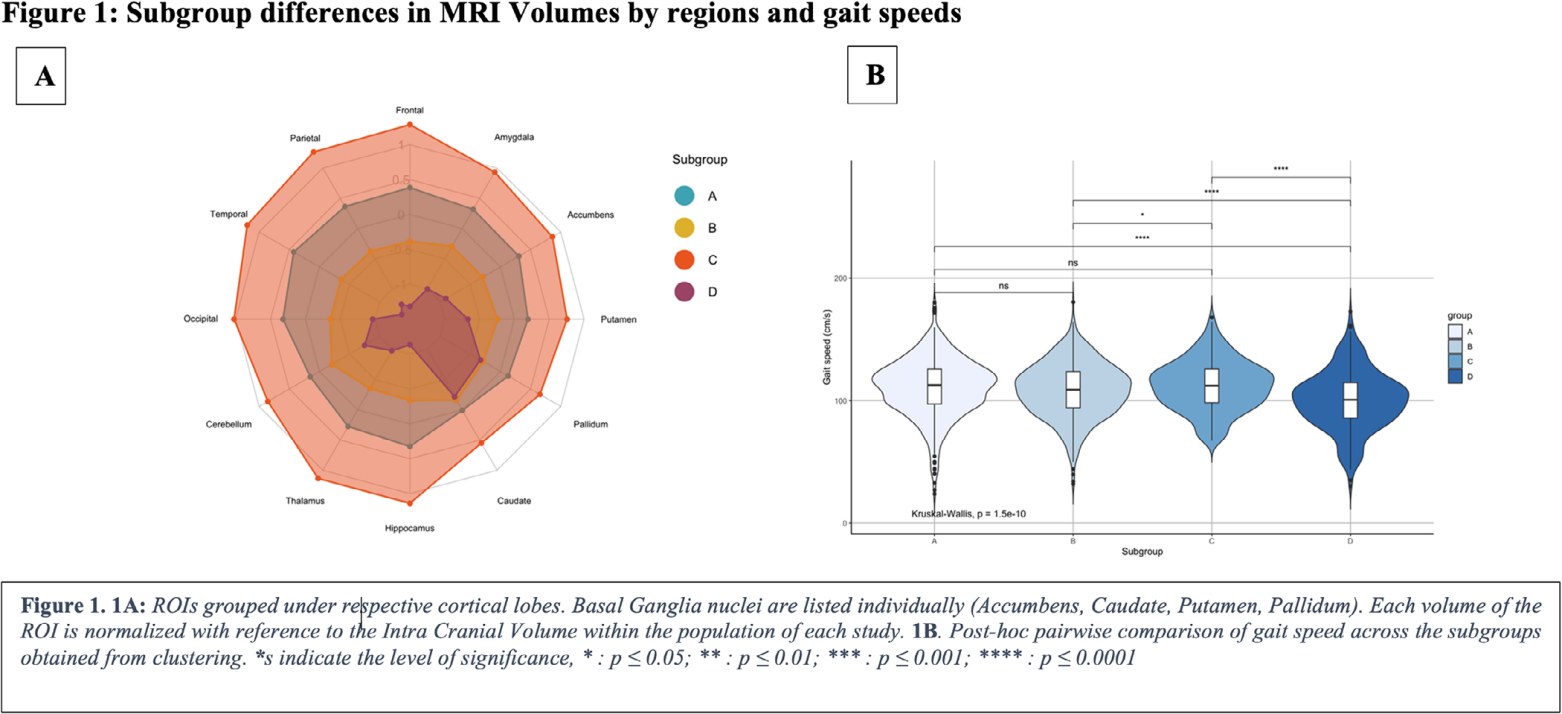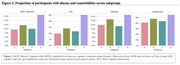# Exploring heterogeneity in Motoric Cognitive Risk Syndrome using Volumetric MRI‐guided Clustering

**DOI:** 10.1002/alz.091117

**Published:** 2025-01-09

**Authors:** Bhargav Teja Nallapu, Helena M Blumen, Kellen K. Petersen, Richard B. Lipton, V.G. Pradeep, Velandai K Srikanth, Richard Beare, Olivier Beauchet, Sofiya Milman, Sandra Aleksic, Ali Ezzati, Joe Verghese

**Affiliations:** ^1^ Albert Einstein College of Medicine, Bronx, NY USA; ^2^ Baby Memorial Hospital, Kozhikode, Kerala India; ^3^ National Centre for Healthy Ageing, Melbourne, VIC Australia; ^4^ Murdoch Children’s Research Institute, Royal Children’s Hospital, Melbourne, VIC Australia; ^5^ McGill University, Montreal, QC Canada; ^6^ University of Montreal, Montreal, QC Canada; ^7^ University of California, Irvine, Irvine, CA USA

## Abstract

**Background:**

The Motoric Cognitive Risk Syndrome (MCR) is a predementia stage characterized by slow gait speed and subjective cognitive complaints. Defining the heterogeneity of brain volumetrics in individuals with MCR will improve current dementia risk assessments.

**Method:**

We used data from 6 cohorts from the MCR consortium (N=2,007). We used K‐means clustering algorithm guided by volumetric MRI to identify distinct subgroups of participants. We compared the differences in cortical and subcortical volumes, comorbidities, and gait speeds across the identified subgroups using one‐way ANOVA and post‐hoc pairwise group comparisons.

**Result:**

The sample had a mean age of 71.89 (±7.05) years, 48.7% were women, 32.9% were White and 63.2% were Asian (see Table 1). Four subgroups (A to D) were identified through MRI‐based clustering with significant differences in brain region volumes (Figure 1A), gait speeds (Figure 1B) and proportion of individuals with MCR (Figure 2). Subgroups A and C had the least amount of atrophy in all brain regions and had the least proportion of MCR. Subgroup D had the highest proportion of MCR subjects and highest atrophy specifically in hippocampus and cortical regions. The average gait speed of Subgroup D was lower than other subgroups. Subgroup D also had the highest rate of hypertension and diabetes among the subgroups (Figure 2).

**Conclusion:**

Our results validate the previous findings linking MCR syndrome to MRI evidence of neurodegeneration. Heterogeneity in cortical and subcortical signatures are present in older adults and provides insights into brain substrates of MCR.